# Fluoroscopy-Guided Percutaneous Transthoracic Needle Lung Biopsy with the Aid of Planning Cone-Beam CT: Diagnostic Accuracy and Complications

**DOI:** 10.3390/diagnostics14212441

**Published:** 2024-10-31

**Authors:** Sang Hyun Cho, Hyun Jung Yoon, Young Lee, Injoong Kim, Je Ryung Gil, Yeo Jin Kim

**Affiliations:** 1 Department of Radiology, Veterans Health Service Medical Center, Seoul 05368, Republic of Korea; briancho2005@naver.com (S.H.C.); kinjoong@gmail.com (I.K.); rlfwpthf@nate.com (J.R.G.); ladefense05@hanmail.net (Y.J.K.); 2 Veterans Medical Research Institute, Veterans Health Service Medical Center, Seoul 05368, Republic of Korea; lyou7688@gmail.com

**Keywords:** lung, biopsy, image-guided biopsy, fluoroscopy, cone-beam computed tomography

## Abstract

**Background:** Fluoroscopy-guided PTNB for fluoroscopy-identifiable lung lesions has been suggested as a useful method for the pathological diagnosis of lung lesions; however, it is lacking in accuracy and safety compared to CT-guided PTNB. Thus, we aimed to investigate the diagnostic accuracy and complications of fluoroscopy-guided percutaneous transthoracic needle biopsy (PTNB) with the aid of pre-procedural planning cone-beam computed tomography (CBCT) in order to take advantage of their respective strengths. **Methods:** A total of 255 fluoroscopy-guided PTNBs with the aid of planning CBCT were performed. Pre-procedural planning CBCT was conducted to calculate the shortest length from the skin puncture site to the margin of the target lesion for the needle trajectory. No intra-procedural CBCT was performed. The diagnostic performance of fluoroscopy-guided PTNB with the aid of planning CBCT was calculated. The prognostic factors for diagnostic failures and complications were evaluated using logistic regression analysis. **Results:** The accuracy, sensitivity, specificity, PPV, and NPV were 97.3%, 88.0%, 90.9%, 100%, and 62.5%, respectively. There were 29 diagnostic failures (11.8%), and the multivariable analysis showed that a longer lesion depth on CBCT and a shorter specimen length were each associated with diagnostic failure (*p* = 0.010 and 0.012, respectively). Complications occurred in 34 PTNBs (13.3%). The multivariable analysis showed that an increased total number of biopsies per lesion, a longer length of lung aeration via needle insertion, a smaller lesion size on CT imaging (≤20 mm), and the presence of an air bronchogram were associated with the occurrence of complications (*p* = 0.027, <0.001, 0.003, and 0.020, respectively). **Conclusions:** Excellent diagnostic accuracy was obtained by fluoroscopy-guided PTNB with the aid of planning CBCT. Compared to that of CT- or CBCT-guided PTNB, the procedure-related complication rate was acceptably low, but the radiation dose to patients could be potentially reduced.

## 1. Introduction

Image-guided percutaneous transthoracic needle biopsy (PTNB) has been widely performed to achieve the pathological diagnosis of lung nodules, and fluoroscopy or conventional computed tomography (CT) guidance is common [1,2,3,4]. In particular, CT-guided PTNB carries advantages in terms of safety and accuracy, but it does not provide real-time guidance and requires long procedure times [1,2,3]. High radiation doses and a small gantry bore limiting the needle length are other critical drawbacks of CT-guided PTNB. In contrast, fluoroscopy-guided PTNB boasts the advantages of real-time guidance, shorter procedure times, lower patient radiation doses, and no limitations to the needle length or imaging plane orientation for needle placement. However, the process is suboptimal in terms of safety and accuracy [5].

Recently, similar diagnostic accuracy to that of conventional CT-guided PTNB and a reduction in patient radiation doses were documented in association with cone-beam CT (CBCT)-guided PTNB [4,6,7,8,9]. Nevertheless, in most studies of CBCT-guided PTNB, both pre- and intra-procedural CBCT were performed; therefore, it remains time-consuming to perform, and the patient radiation exposure is high compared to that seen with fluoroscopy-guided PTNB [9,10]. In this study, we performed fluoroscopy-guided PTNB for fluoroscopy-identifiable lung lesions with only planning CBCT (i.e., without intra-procedural CBCT), and we evaluated whether the accuracy and safety of the procedure could be maintained while potentially reducing the radiation exposure among patients.

Thus, the purpose of this study was to investigate the diagnostic accuracy and complications of fluoroscopy-guided PTNB with the aid of pre-procedural planning CBCT and to evaluate the prognostic factors for diagnostic failure and complications.

## 2. Materials and Methods

### 2.1. Study Design and Population

This retrospective study was approved by the Institutional Review Board (IRB) of the Veterans Health Service Medical Center, and the requirement for written informed consent was waived (IRB no. 2023-03-010).

A total of 309 consecutive patients underwent image-guided PTNB in our hospital between January 2018 and December 2023. Of these patients, 64 who underwent only fluoroscopy-guided (60 patients) or CT-guided (four patients) PTNBs were excluded. Only fluoroscopy-guided PTNB was performed when the target lesion was subpleurally located, had little change in lesion depth (i.e., the shortest length from the skin of the puncture site to the margin of the target lesion for needle trajectory) according to position changes or respiratory correction, and was not adjacent to large vascular structures or airways. CT-guided PTNB was performed when the target lesion could not be identified or was poorly delineated under fluoroscopic guidance. Finally, we included 245 patients who satisfied the selection criterion regarding the target lesion in this study. Among these 245 patients, nine underwent repeat biopsies for the same target lesion. One of these nine patients underwent three PTNB sessions, while the others each underwent two PTNB sessions. The median number of days between the initial and repeat procedures was nine. All repeat PTNBs were included in our study because there was no clinical interference between the procedures—that is, there were no post-procedural complications after the initial PTNB. Finally, 255 PTNBs in 245 patients were included in the study. With respect to the diagnostic performance analysis, 10 PTNBs that did not fulfill the predefined criteria for the final diagnosis were excluded.

### 2.2. Fluoroscopy-Guided PTNB with Planning CBCT

Fluoroscopy-guided PTNBs with the aid of planning CBCT were performed by a thoracic radiologist with 10 years of experience in image-guided PTNB. The PTNBs were performed using a CBCT (Axiom Artis zee biplane/AS40 flat-panel detector with a 2480 × 1920 element (Siemens, Forchheim, Germany) or Allura Xper FD2015 flat-panel detector with a 2586 × 1904 element (Philips Healthcare, Best, The Netherlands) with needle trajectory-planning software (iGuide syngoXWP VB21N (Siemens) or XperGuide 1.1.11 (Philips Healthcare)) and real-time fluoroscopy.

All procedures were performed after a review of the pre-procedural contrast-enhanced chest CT scans collected during the diagnostic assessment. The sequential steps for PTNB were as follows: depending on the location of the target lesion, the patient was placed in either a prone or supine position; then, under the guidance of real-time fluoroscopy, with the patient in the correct respiration state, the appropriate skin entry site was visually determined and marked on the overlying skin. Next, pre-procedural planning CBCT was performed to evaluate the shortest length from the skin entry site to the margin of the target lesion to ascertain the safest and most effective needle pathway to the target lesion. After determining the optimal needle pathway to the target lesion, the skin entry site was disinfected, and careful local anesthesia was conducted. An 18- or 20-gauge gun biopsy needle with an active needle part (tip) of 11, 16, or 22 mm in length (Gunbiopsy; TSK Laboratory, Tochigi, Japan) was inserted into the target lesion under real-time fluoroscopy guidance until it was positioned correctly. During the progression of the needle and biopsy sampling, the patient was asked to stop breathing. The adequacy of the biopsy specimen was visually assessed by the operator, and another satisfactory specimen was obtained if necessary. Rapid on-site cytopathology examination [11] was not available at our unit. Biopsy sampling was performed no more than three times. Post-procedural fluoroscopy acquisition was conducted to identify procedure-related complications. Immediately after PTNB and during the hospital stay, follow-up chest radiography was performed to identify procedure-related complications. If a patient developed minor asymptomatic pneumothorax or hemothorax, the treatment was conservative with oxygen. When patients developed symptomatic pneumothorax, a chest tube was inserted for symptomatic relief.

### 2.3. Data Collection

One thoracic radiologist with 10 years of clinical experience retrospectively reviewed the pre-procedural CT images of the PTNBs using a picture-archiving and communication system station. On the axial scans of the pre-procedural contrast-enhanced chest CT and planning CBCT, the shortest length from the skin entry site to the margin of the target lesion, as the safest and most effective needle pathway to the target lesion, was measured, and the difference in the lengths measured between diagnostic CT and CBCT was recorded. The length of lung aeration via needle insertion was also measured and recorded.

Variables related to the patients, target lesions, and biopsy procedures were collected from the PTNB data registry at our hospital. The patient variables included age; sex; underlying lung diseases, including pulmonary emphysema, interstitial lung disease, or pneumoconiosis, on pre-procedural diagnostic CT images; and a history of previous lung operations. The target lesion characteristics included the lesion size (maximal diameter on a diagnostic CT image), location, lesion type (nodule or mass, consolidation), pleural contact, peribronchovascular location, air bronchogram, cavity or necrosis, and solidity. The biopsy-associated variables of interest were the patient position, biopsy needle, number of tissue samples per lesion, and specimen length.

Complications including pneumothorax, hemoptysis, and cough with blood oxygen deceleration were recorded after reviewing the procedure records, medical charts, and follow-up images. Complications requiring additional procedures (i.e., those with a Clavien–Dindo classification ≥II) were also recorded using the same method [12]. The patient radiation doses during fluoroscopy and CBCT during each PTNB procedure were recorded in milligray (mGy) units. We registered the procedure time, defined as the time from the skin marking of the entry site to the postprocedural radiograph. The fluoroscopy time was also recorded.

Based on the pathological report on the biopsy specimen, the PTNB results were classified as positive, negative, or non-evaluable due to insufficient specimens, and the final diagnosis was blinded [9]. Based on previous research, malignancies and atypical cells suspected of being malignant were regarded as positive [9,12]. In contrast, specific benign diagnoses such as hamartoma and non-specific benign pathology results such as granulomatous inflammation or organizing pneumonia were regarded as negative [8,9,12]. Lastly, the results were regarded as non-evaluable if the specimens were insufficient or unsuitable for diagnosis due to a lack of cells [9].

### 2.4. Reference Standards

The reference standard of malignancy or benignity for each lesion was set in the following ways, according to the methods suggested by Hong et al. [2,7,9]. (1) If the target lesion was surgically resected, the final diagnosis was based on the surgical pathology report. (2) For target lesions that were not surgically resected, the final diagnosis was based on pathologic analysis of the PTNB. The results of atypical cells were not included in the final diagnosis. (3) The target lesion was regarded as benign if it decreased in size by more than 20% or remained stable for more than 2 years without treatment. (4) Lesions were judged to be malignant if they exhibited clinically malignant behavior. Lesions that did not meet the above criteria were categorized as incomplete reference standards and were excluded from the diagnostic performance analysis.

### 2.5. Statistical Analysis

The diagnostic performance measures of accuracy, sensitivity, specificity, positive predictive value (PPV), and negative predictive value (NPV) were calculated with 95% confidence intervals (CIs) for the detection of malignancies on a per-lesion basis. According to the intention to diagnose principle, non-evaluable results due to insufficient specimen collection were treated as false negatives for sensitivity calculations and false positives for specificity calculations [8,9,13].

Diagnostic success after PTNB was marked by true-positive and true-negative results. Conversely, diagnostic failures were marked by either false-positive, false-negative, or non-evaluable results. To determine the prognostic factors for diagnostic failure, we performed univariable and multivariable logistic regression using Firth’s penalized maximum likelihood bias reduction method. The Firth method is a general approach to reducing small-sample bias in maximum likelihood estimation [14,15]. All variables with a *p* < 0.10 in the univariable analyses were included, and backward variable selection was conducted. The rates of overall complications and of each type of complication were calculated. The prognostic factors for overall complications were investigated using the same method. The prognostic factors for the difference in the shortest length from the skin entry site to the margin of the target lesion between CT and planning CBCT were analyzed using the same method.

All statistical analyses were performed using the R software, version 4.0.1 (R Foundation for Statistical Computing, Vienna, Austria; http://www.R-project.org accessed on 10 January 2024). *p* < 0.05 was considered to indicate statistical significance.

## 3. Results

### 3.1. Patient Demographics and Lesions

In total, 245 patients underwent 255 fluoroscopy-guided PTNBs with the aid of planning CBCT, and their mean age was 75.8 ± 6.1 years (range, 43–93 years). After excluding 10 cases with incomplete reference standards, the diagnostic performance of 245 fluoroscopy-guided PTNBs with the aid of planning CBCT in 237 patients with a mean age of 75.7 ± 6.2 years (range, 43–93 years) was analyzed. The characteristics of the patients, lesions, and procedures are shown in Table 1. The average radiation dose to patients was estimated to be 173.7 ± 112.6 mGy (range, 11.0–728.4 mGy). The mean total procedure time and fluoroscopy time were 11.1 ± 3.0 min (range 6–26 min) and 2.2 ± 0.8 min (range 1.1–4.8 min), respectively.

### 3.2. Diagnostic Accuracy

Among the 245 PTNBs, 206 (84.1%) showed positive results, 16 (6.5%) showed negative results, and 23 (9.4%) were non-evaluable. According to the final diagnosis based on the reference standard, 234 (95.5%) lesions were confirmed to be malignant, while 11 (4.5%) lesions were confirmed to be benign. The final diagnosis of a malignancy was based on the surgical pathology (*n* = 65), specific malignant biopsy results (*n* = 162), and clinically malignant behavior (*n* = 7). Benignity was confirmed based on the specific benign biopsy results (*n* = 7) or a significant decrease or stability in size for ≥2 years (*n* = 4). Thus, 65 of 245 PTNBs (26.5%) were confirmed by surgical resection after PTNB.

The overall accuracy, sensitivity, specificity, PPV, and NPV for the diagnosis of malignancies were 97.3% (216/222; 95% CI, 94.2–99.0%), 88.0% (206/234; 95% CI, 83.2–91.9%), 90.9% (10/11; 95% CI, 58.7–99.8%), 100% (216/216; 95% CI, 98.2–100%), and 62.5% (10/16; 95% CI, 35.4–84.8%), respectively (Table 2).

Of note, 192 specimens were ultimately diagnosed with NSCLC, with 63 in stage I, 28 in stage II, 34 in stage III, and 67 in stage IV. Molecular analysis for lung cancer drug therapy was performed on 105 specimens through PTNB, of which 91 (86.7%) were successful.

### 3.3. Diagnostic Failure and Prognostic Factors

**Figure 1:**
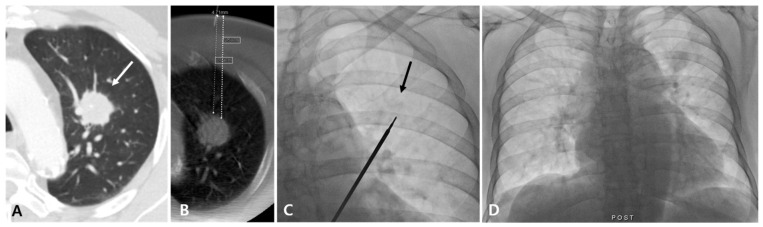
Diagnostic failure of fluoroscopy-guided PTNB with the aid of planning CBCT for a lung nodule in a 78-year-old man with lung adenocarcinoma. A diagnostic CT image (**A**) and planning CBCT image (**B**) show a 28 mm nodule (white arrow) in the left upper lobe with a long lesion depth (85 mm, bold white dashed line). On fluoroscopy, the nodule is identifiable (black arrow) (**C**) and no immediate PTNB-related complications occurred (**D**). The pathologic specimen length of the lesion was 1 mm, and the pathology result was non-evaluable in this case.

The diagnostic failure group included 29 (11.8%) PTNBs. Table 3 summarizes the results of the analysis comparing the diagnostic success and failure groups. The univariable logistic regression analysis revealed that the patient position, biopsy needle caliber and active needle part length, lesion depth on CBCT imaging, specimen length, lesion type, and peribronchovascular location could be used as input variables for the multivariable analysis (*p* = 0.049, 0.033, 0.019, 0.011, 0.051, and 0.016, respectively). The multivariable logistic regression analysis revealed that a longer lesion depth on CBCT imaging and a shorter specimen length were associated with diagnostic failure (*p* = 0.010 and 0.012, respectively) (Figure 1).

### 3.4. PTNB-Related Complications

PTNB-related complications occurred after 34 (13.3%) of the 255 PTNBs. In particular, pneumothorax occurred after 20 PTNBs (7.8%), with significant pneumothorax requiring drainage catheter insertion occurring after three PTNBs (1.2%). Hemoptysis occurred after seven (2.7%) of the PTNBs. Other complications included cough and a blood oxygen saturation decrease after six (2.4%) PTNBs and bradycardia after one (0.4%) PTNB, all of which resolved without additional procedures. There were no cases of abdominal solid organ injuries, air embolism, or mortality. The major complication rate was 1.2%.

**Figure 2:**
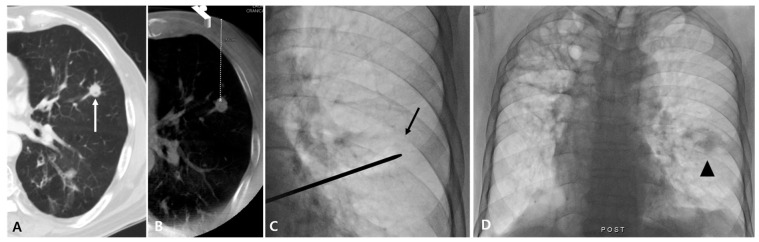
Case of fluoroscopy-guided PTNB with the aid of planning CBCT of a lung nodule in a 79-year-old man with lung metastatic adenocarcinoma that resulted in a complication. A diagnostic CT image (**A**) and planning CBCT image (**B**) show an 11 mm nodule (white arrow) in the left upper lobe with a long aerated lung length achieved by needle insertion (46 mm). A lesion depth was 75.6 mm (white dashed line). On fluoroscopy, the nodule is identifiable (black arrow) (**C**), and PTNB was performed twice. After PTNB, hemoptysis occurred, and a post-PTNB fluoroscopic image shows peritumoral consolidation, representing a hemorrhage (arrowhead) (**D**).

Regarding the prognostic factors for complications, the univariable logistic regression analysis revealed the total number of biopsies per lesion, lesion depth on CBCT, length of lung aeration via needle insertion, lesion size on CT, pleural contact, and air bronchogram as input variables for the multivariable analysis (*p* = 0.057, <0.001, <0.001, 0.014, 0.003, and 0.068, respectively). The multivariable logistic regression analysis revealed that an increased total number of biopsies per lesion, longer length of lung aeration by needle insertion, smaller lesion size on CT (≤20 mm), and air bronchogram were associated with the occurrence of complications (*p* = 0.027, <0.001, 0.003, and 0.020, respectively) (Figure 2). Table 4 summarizes the results of the univariable and multivariable analyses for the prognostic factors of complications.

### 3.5. Difference between Lesion Depths Measured on Diagnostic CT and CBCT Scans

**Figure 3:**
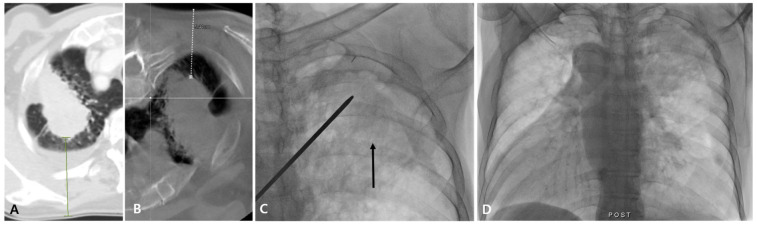
A >10 mm difference in the lung nodule lesion depth was observed via fluoroscopy-guided PTNB with the aid of planning CBCT in a 75-year-old man with lung adenocarcinoma. A diagnostic CT image (**A**) shows a 69 mm lesion depth (green line), and a planning CBCT image in the prone position with scapular lateral rearrangement (**B**) shows a 54 mm lesion depth (white dashed line) for a 70 mm mass in the left apex. On fluoroscopy, the mass is identifiable (arrow) (**C**), and PTNB was performed based on the 54 mm lesion depth observed via CBCT. After PTNB, no immediate PTNB-related complications occurred (**D**), and diagnostic success was achieved.

The average lesion depths measured on the diagnostic CT and CBCT scans for all 255 PTNBs were 52.9 ± 19.9 mm (range, 13.7–121.2 mm) and 51.4 ± 18.5 mm (range, 16.2–103.0 mm), respectively. The average difference between the lesion depths measured on the diagnostic CT and CBCT scans for the same target lesion was 7.5 ± 6.5 mm (range, 0.1–38.5 mm). As for the difference in the lesion depths measured on the diagnostic CT and CBCT scans, the univariable and multivariable logistic regression analyses revealed that a history of previous lung operations and an apex-located lesion were prognostic factors for a difference ≥10 mm in lesion depth (*p* = 0.015 and <0.001, respectively) (Table 5) (Figure 3).

## 4. Discussion

In this study, we evaluated the diagnostic accuracy of fluoroscopy-guided PTNB with the aid of planning CBCT and its related complications. The accuracy for the diagnosis of malignancies was 97.3%; the sensitivity and specificity were acceptable at 88.0% and 90.9%, respectively; and diagnostic failure occurred after 7.5% of the procedures in this study, which is equivalent to or slightly higher than the rates reported for conventional CT-guided or CBCT-guided PTNB in previous studies [1,2,3,6,8,9,10,16,17,18]. Importantly, CBCT was performed only once, when the entry site was marked on the site of the overlying skin; only fluoroscopy guidance was used during targeting and tissue sampling and immediately after the PTNB to identify procedure-related complications. This approach may reduce the patient’s radiation exposure compared to that when using other CBCT-guided PTNB approaches reported in the literature, which exhibit a mean patient radiation dose ranging from 170.0 to 567.5 mGy [4,9,19], based on the radiation exposure in our study (173.7 ± 112.6 mGy). Despite the observed reductions in the patient radiation dose, we believe that the diagnostic accuracy seen in this study is reasonable for clinical situations where a biopsy is required to differentiate malignant tumors from benign lesions.

However, as mentioned in the Materials and Methods section, we performed a CT-guided biopsy if a lesion was not clearly visualized on the fluoroscopic images. Moreover, target lesions that underwent fluoroscopy-guided biopsy only were excluded from CBCT-aided fluoroscopic biopsy if they were subpleurally located, had little change in lesion depth according to position changes or respiratory correction, and no adjacent large vascular structure or airway was present. These exclusions could have made it more difficult to compare the accuracy and risks of complications in this study with those reported in other CT- or CBCT-guided PTNB studies. Thus, the high accuracy and safety of fluoroscopy-guided PTNB with the aid of planning CBCT can be achieved when adopting rigorous inclusion criteria according to the findings on CT imaging.

As for the prognostic factors for diagnostic failure, a longer lesion depth on CBCT and a shorter specimen length were both significantly associated with diagnostic failure in this study. It is widely known that longer specimen lengths are associated with greater diagnostic accuracy and that more deeply located lung lesions are more difficult to target accurately using biopsy needles. The finding that a large needle caliber and long active needle part length were significant in the univariable analysis but not the multivariable analysis seems to be associated with the acquisition of longer and thicker samples.

We believe that the performance of planning CBCT when the entry site has been marked on the chest wall could yield more accurate information regarding the needle trajectory and lesion depth than diagnostic CT. Planning CBCT images could depict potential alterations in the needle trajectory while traversing the chest wall, because there is a possibility for alterations in the lesion location with a change in the patient position (e.g., supine to prone, arm position change) or respiratory correction (diagnostic CT: full inspiration vs. CBCT for PTNB: variable due to adjustments to avoid intra-costal lesion positioning) compared to diagnostic chest CT. We believe that this hypothesis is supported by the apex lesion location as a predictor of a greater difference between the lesion depths measured on the diagnostic CT and CBCT scans, because there may have been large differences in the chest wall thickness due to scapula rearrangement when the patient was prone (Figure 3). A history of lung operations was also an associated prognostic factor in this study. Presumably, major thoracic surgery can cause fibrotic changes in the lung, pleura, and chest wall, which lead to reduced flexibility and thus cause large movements of the lesion according to posture changes. In such situations, the real needle trajectory may deviate substantially from the pathways displayed on the diagnostic CT scan.

In this study, complications occurred after 13.3% of the fluoroscopy-guided PTNBs performed with the aid of planning CBCT for fluoroscopy-identifiable lung lesions, which was near the lower end of the range reported for complications after PTNB (12.1–49.8%) [3,4,5,6,9,16,17,19,20,21]. In particular, pneumothorax requiring drainage occurred in just 1.2% of cases, and this rate was lower than those reported for conventional CT, fluoroscopy CT, and CBCT-guided PTNB, which collectively ranged from 0.9 to 7.0% [3,4,5,6,9,10,16,17]. We believe that the accurate information regarding the needle trajectory provided by the planning CBCT could have reduced the potential occurrence of complications in this study.

As for the prognostic factors for complications, we concluded that multiple biopsies per lesion, a longer length of lung aeration via needle insertion, and the presence of an air bronchogram were related to the occurrence of complications. It is already known that an air bronchogram is associated with hemoptysis [22]. It is not clearly understood why a smaller lesion size on diagnostic CT imaging is associated with the occurrence of complications, but it may be related to the longer fluoroscopic times required to ensure accuracy [4,13].

The following are potential limitations of this study. First, this was a single-center study, and further large-scale multicenter studies will enhance the strength of our findings. Second, the number of benign lung nodules was too small to draw strong conclusions about the specificity or NPVs. Third, the rate of non-evaluable PTNBs due to inadequate specimens was 10.2%, which was higher than the rates in the literature [9,23]. We believe that this might be explained by the poor respiration control in elderly patients with underlying lung diseases, such as chronic bronchitis or emphysema. Fourth, the 90.9% specificity of PTNB reported in our study was lower than previously reported values, which range from 98 to 100% [2,3,6,7,16,17]. This discrepancy might be explained by the identification of non-evaluable results on PTNB as false positives that were confirmed to be benign based on the reference standards. In previous studies, such results were excluded or regarded as negative [2,3,6,7,16,17]. Nevertheless, the specificity in this study was very similar to that (91.4%) in a study of CBCT-guided PTNB abiding by the same principles [9]. Finally, immediate post-procedural CT may be necessary to detect serious complications such as air embolism, but, in our study, we did not perform CT and only used post-procedural fluoroscopy and chest radiographs to identify complications. However, we believe that our study shows that this procedure can be performed relatively safely and accurately even in situations where CT is not available or preferred. Nevertheless, care should be taken to avoid air embolism.

In conclusion, when performed after fulfilling rigorous criteria, fluoroscopy-guided PTNB aided by planning CBCT provided excellent diagnostic accuracy for malignancies, comparable to that reported in previous studies for conventional CT or dual (planning and intra-procedural) CBCT-guided PTNB, and the procedure-related complication rate was acceptably low compared to the rates in the same previous studies. However, the patient radiation dose could still be reduced.

## Figures and Tables

**Table 1:** Characteristics of patients, lesions, and procedures.

Variable	Analysis of Diagnostic Performance	Analysis of Complications
Total no. of procedures	245	255
Total no. of patients	237	245
Age	75.7 ± 6.2	75.8 ± 6.1
Sex		
Male–female	224 (91.4):21 (8.6)	232 (91.0):23 (9.0)
Duration between CT and CBCT (days)	15.3 ± 13.0	15.1 ± 12.9
Position		
Supine–prone	86 (35.1):159 (64.9)	88 (34.5):167 (65.5)
Biopsy needle		
18-G, 16 mm tip	181 (73.9)	185 (72.5)
20-G, 22 mm tip	56 (22.9)	62 (24.3)
20-G, 11 mm tip	3 (1.2)	3 (1.2)
18-G, 11 mm tip	5 (2.0)	5 (2.0)
Total number of biopsies per lesion		
1	181 (73.9)	188 (73.7)
2	58 (23.7)	61 (23.9)
3	6 (2.4)	6 (2.4)
Radiation dose (mGy)	169.5 ± 110.0	173.7 ± 112.6
Lesion depth on CT (mm) *	52.4 ± 19.3	52.9 ± 19.9
Lesion depth on CBCT (mm) *	51.1 ± 18.4	51.4 ± 18.5
Difference in lesion depth between CT and CBCT (mm)	7.3 ± 6.5	7.5 ± 6.5
Length of lung aeration via needle insertion (mm)	17.7 ± 16.7	17.6 ± 16.5
Specimen length (mm)	8.2 ± 3.6	8.2 ± 3.6
Pathological results		
Positive	206 (84.1)	210 (82.4)
Negative	16 (6.5)	19 (7.5)
Non-evaluable	23 (9.4)	26 (10.2)
Final diagnosis		
Benign	11 (4.5)	11 (4.3)
Malignant	234 (95.5)	234 (91.8)
Incomplete		10 (3.9)
History of lung operation, yes	6 (2.4)	6 (2.4)
Underlying lung disease, yes	123 (50.2%)	124 (48.6%)
Lesion size on CT (mm)	36.7 ± 20.8	36.7 ± 21.4
Lesion location		
Apex	14 (5.7)	15 (5.9)
Upper	96 (39.2)	97 (38.0)
Middle	29 (11.8)	31 (12.2)
Lower	106 (43.3)	112 (43.9)
Lesion type		
Nodule or mass	226 (92.2)	234 (91.8)
Consolidation	19 (7.8)	21 (8.2)
Pleural contact, yes	114 (46.5)	120 (47.1)
Peribronchovascular location, yes	37 (15.1)	39 (15.3)
Air bronchogram, yes	16 (6.5)	18 (7.1)
Necrosis, yes	53 (21.6)	54 (21.2)
Cavity, yes	18 (7.3)	19 (7.5)
Solidity		
Solid–subsolid	235 (95.9):10 (4.1)	245 (96.1):10 (3.9)
Complication, yes	32 (13.1)	34 (13.3)

Data are presented as mean ± standard deviation. Unless otherwise indicated, data in parentheses are percentages. * Lesion depth is defined as the shortest length from the skin of the puncture site to the margin of the target lesion for needle trajectory. *Abbreviations:* CBCT, cone-beam computed tomography; CT, computed tomography; G, gauge.

**Table 2:** Diagnostic performance of fluoroscopic-guided PTNB with planning CBCT.

	Total No. of Procedures		Estimate (95% CI)
True positive	206	Accuracy	97.3 (94.2–99.0)
True negative	10	Sensitivity	88.0 (83,2–91.9)
False positive	0	Specificity	90.9 (58.7–99.8)
False negative	6	PPV	100.0 (98.2–100.0)
Non-evaluable	23 (22 malignancy + 1 benign)	NPV	62.5 (35.4–84.8)

*Abbreviations:* CI, confidence interval; PPV, positive predictive value; NPV, negative predictive value.

**Table 3:** Results of univariable and multivariable analyses of prognostic factors for the diagnostic failure of fluoroscopic-guided PTNB with planning CBCT (*n* = 245).

Variable	Diagnostic Success (*n* = 216)	Diagnostic Failure (*n* = 29)	Univariable Analysis	Multivariable Analysis	
			*p* Value	Odds Ratio (95% CI)	*p* Value
Age	75.5 ± 6.2	77.1 ± 5.4	0.209		
Sex, male	199 (92.1)	25 (86.2)	0.244		
Position, prone	145 (67.1)	14 (48.3)	0.049		
Biopsy needle			0.033		
18-G, 16 mm tip	164 (75.9)	17 (58.6)			
20-G, 22 mm tip	46 (21.3)	10 (34.5)			
20-G, 11 mm tip	1 (0.5)	2 (6.9)			
18-G, 11 mm tip	5 (2.3)	0 (0.0)			
Total number of biopsies per lesion			0.141		
1	163 (75.5)	18 (62.1)			
2	48 (22.2)	10 (34.5)			
3	5 (2.3)	1 (3.4)			
Lesion depth on CBCT (mm) *	50.1 ± 17.9	58.7 ± 19.9	0.019	1.03 (1.01–1.05)	0.010
Length of lung aeration via needle insertion (mm)	17.2 ± 16.4	20.8 ± 18.2	0.273		
Specimen length (mm)	8.4 ± 3.4	6.6 ± 4.9	0.011	0.87 (0.76–0.97)	0.012
History of lung operation, yes	5 (2.3)	1 (3.4)	0.483		
Underlying lung disease, yes	110 (50.9)	13 (44.8)	0.543		
Lesion size on CT			0.917		
≤20 mm	41 (19.0)	5 (17.2)			
>20 mm	175 (81.0)	24 (82.8)			
Lesion location			0.868		
Apex	12 (5.6)	2 (6.9)			
Upper	83 (38.4)	13 (44.8)			
Middle	26 (12.0)	3 (10.3)			
Lower	95 (44.0)	11 (37.9)			
Lesion type			0.051		
Nodule or mass	202 (93.5)	24 (82.8)			
Consolidation	14 (6.5)	5 (17.2)			
Pleural contact, yes	102 (47.2)	12 (41.4)	0.566		
Peribronchovascular location, yes	28 (13.0)	9 (31.0)	0.016		
Air bronchogram, yes	14 (6.5)	2 (6.9)	0.744		
Necrosis, yes	46 (21.3)	7 (24.1)	0.662		
Cavity, yes	16 (7.4)	2 (6.9)	0.889		
Solidity, subsolid	8 (3.7)	2 (6.9)	0.316		
Final diagnosis, benign	10 (4.6)	1 (3.4)	0.969		

Data are presented as mean ± standard deviation. Unless otherwise indicated, data in parentheses are percentages. * Lesion depth is defined as the shortest length from the skin of the puncture site to the margin of the target lesion for needle trajectory. *Abbreviations:* CBCT, cone-beam computed tomography; CI, confidence interval; CT, computed tomography; G, gauge.

**Table 4:** Results of univariable and multivariable analyses of prognostic factors for complications after fluoroscopic-guided PTNB with planning CBCT (*n* = 255).

Variable	Complications		Univariable Analysis	Multivariable Analysis	
	No (*n* = 221)	Yes (*n* = 34)	*p* Value	Odds Ratio (95% CI)	*p* Value
Age	75.9 ± 6.0	74.9 ± 7.3	0.386		
Sex, male	201 (91.0)	31 (91.2)	0.886		
Position, prone	149 (67.4)	18 (52.9)	0.100		
Biopsy needle			0.534		
18-G, 16 mm tip	162 (73.3)	23 (67.6)			
20-G, 22 mm tip	52 (23.5)	10 (29.4)			
20-G, 11 mm tip	2 (0.9)	1 (2.9)			
18-G, 11 mm tip	5 (2.3)	0 (0.0)			
Total number of biopsies per lesion			0.057	2.14 (1.09–4.20)	0.027
1	167 (75.6)	21 (61.8)			
2	50 (22.6)	11 (32.4)			
3	4 (1.8)	2 (5.9)			
Lesion depth on CBCT (mm) *	49.5 ± 17.8	63.5 ± 18.4	<0.001		
Length of lung aeration via needle insertion (mm)	15.9 ± 16.1	28.9 ± 14.3	<0.001	1.05 (1.03–1.08)	<0.001
Specimen length (mm)	8.3 ± 3.5	7.6 ± 3.8	0.298		
History of lung operation, yes	6 (2.7)	0 (0.0)	0.583		
Underlying lung disease, yes	109 (49.3)	15 (44.1)	0.579		
Lesion size on CT			0.014	0.26 (0.11–0.62)	0.003
≤20 mm	37 (16.7)	12 (35.3)			
>20 mm	184 (83.3)	22 (64.7)			
Lesion location			0.721		
Apex	13 (5.9)	2 (5.9)			
Upper	81 (36.7)	16 (47.1)			
Middle	28 (12.7)	3 (8.8)			
Lower	99 (44.8)	13 (38.2)			
Lesion type			0.748		
Nodule or mass	203 (91.9)	31 (91.2)			
Consolidation	18 (8.1)	3 (8.8)			
Pleural contact, yes	112 (50.7%)	8 (23.5)	0.003		
Peribronchovascular location, yes	32 (14.5%)	7 (20.6)	0.321		
Air bronchogram, yes	13 (5.9%)	5 (14.7)	0.068	3.45 (1.28–14.41)	0.020
Necrosis, yes	45 (20.4%)	9 (26.5)	0.384		
Cavity, yes	19 (8.6)	0 (0.0)	0.986		
Solidity, subsolid	8 (3.6%)	2 (5.9)	0.402		

Data are presented as mean ± standard deviation. Unless otherwise indicated, data in parentheses are percentages. * Lesion depth is defined as the shortest length from the skin of the puncture site to the margin of the target lesion for needle trajectory. *Abbreviations:* CBCT, cone-beam computed tomography; CI, confidence interval; CT, computed tomography; G, gauge.

**Table 5:** Results of univariable and multivariable analyses of prognostic factors for the difference in lesion depth between diagnostic CT and planning cone-beam CT (*n* = 255).

Variable	Difference in Lesion Depth *		Univariable Analysis	Multivariable Analysis	
	<10 mm (*n* = 188)	≥10 mm (*n* = 67)	*p* Value	Odds Ratio (95% CI)	*p* Value
Age	75.6 ± 6.1	76.2 ± 6.4	0.519		
Sex, male	168 (89.4)	64 (95.5)	0.143		
Duration between CT and CBCT (days)	14.6 ± 12.6	16.6 ± 13.7	0.271		
Position, prone	120 (63.8)	47 (70.1)	0.361		
Length of lung aeration via needle insertion (mm)	16.5 ± 16.2	20.8 ± 16.9	0.067		
History of lung operation, yes	2 (1.1)	4 (6.0)	0.033	7.01 (1.48–41.80)	0.015
Underlying lung disease, yes	91 (48.4)	33 (49.3)	0.904		
Lesion size on CT			0.423		
≤20 mm	34 (18.1)	15 (22.4)			
>20 mm	154 (81.9)	52 (77.6)			
Lesion location			<0.001		<0.001
Apex	3 (1.6)	12 (17.9)		13.17 (3.94–55.82)	
Upper	75 (39.9)	22 (32.8)		Reference	
Middle	26 (13.8)	5 (7.5)		0.66 (0.21–1.82)	
Lower	84 (44.7)	28 (41.8)		1.01 (0.52–1.98)	
Lesion type			0.804		
Nodule or mass	173 (92.0)	61 (91.0)			
Consolidation	15 (8.0)	6 (9.0)			
Pleural contact, yes	88 (46.8)	32 (47.8)	0.893		
Peribronchovascular location, yes	28 (14.9)	11 (16.4)	0.767		
Air bronchogram, yes	16 (8.5)	2 (3.0)	0.100		
Necrosis, yes	37 (19.7)	17 (25.4)	0.334		
Cavity, yes	15 (8.0)	4 (6.0)	0.583		
Solidity, subsolid	8 (4.3)	2 (3.0)	0.636		

Data are presented as mean ± standard deviation. Unless otherwise indicated, data in parentheses are percentages. * Lesion depth is defined as the shortest length from the skin of the puncture site to the margin of the target lesion for needle trajectory. *Abbreviations:* CBCT, cone-beam computed tomography; CI, confidence interval; CT, computed tomography.

## Data Availability

The data presented in this study are available upon request from the corresponding author.
